# Anti-inflammatory effects of methoxyphenolic compounds on human airway cells

**DOI:** 10.1186/1476-9255-9-6

**Published:** 2012-03-13

**Authors:** Kenneth R Houser, David K Johnson, Faoud T Ishmael

**Affiliations:** 1 Departments of Medicine and Biochemistry and Molecular Biology, The Pennsylvania State University College of Medicine, Hershey, PA 17033, USA; 2 Department of Chemistry, State University of New York College at Geneseo, Geneseo, NY 14454, USA

**Keywords:** Cytokines, Inflammation, Airway, Epithelium, Methoxyphenols, Post-transcriptional regulation

## Abstract

**Background:**

The respiratory epithelium plays a central role in the inflammatory response in asthma and other diseases. Methoxyphenolic compounds are purported to be effective anti-inflammatory agents, but their effects on the airway epithelium have not been well characterized.

**Methods:**

Human airway cells were stimulated with TNF-α in the presence or absence of 4-substituted methoxyphenols and resveratrol. The expression of various cytokines was measured by qPCR, ELISAs, and protein arrays. Reactive oxygen species (ROS) production was measured with a reactive fluorescent probe (3',6'-diacetate-2',7'-dichlorofluorescein). Activation of NF-κB was measured by nuclear translocation and phosphorylation. Ribonuclear protein association with mRNA was assessed with a biotin-RNA affinity isolation assay.

**Results:**

Multiple inflammatory mediators were inhibited by methoxyphenols, including: CCL2, CCL5, IL-6, IL-8, ICAM-1, MIF, CXCL1, CXCL10, and Serpin E1. IC_50 _values were obtained for each compound that showed significant anti-inflammatory activity: diapocynin (20.3 μM), resveratrol (42.7 μM), 2-methoxyhydroquinone (64.3 μM), apocynin (146.6 μM), and 4-amino-2-methoxyphenol (410 μM). The anti-inflammatory activity did not correlate with inhibition of reactive oxygen species production or NF-κB activation. However, methoxyphenols inhibited binding of the RNA-binding protein HuR to mRNA, indicating that they may act post-transcriptionally.

**Conclusions:**

Methoxyphenols demonstrate anti-inflammatory activity in human airway cells. More potent compounds that act via similar mechanisms may have therapeutic potential as novel anti-inflammatory agents.

## Background

The airway epithelium plays a crucial role in the pathogenesis of asthma, chronic obstructive pulmonary disease, and other inflammatory lung diseases. By serving as a key interface between the host and environment, the airway participates in activation of innate immunity and production of cytokines and other inflammatory mediators in response to microbes, allergens, pollutants, and other inflammatory stimuli [1,2]. As such, the airway epithelium is a prime target for anti-inflammatory agents.

The production of cytokines from the airway epithelium has been shown to play an important role in regulating inflammation associated with respiratory diseases. In response to various stimuli, the airway epithelium produces numerous inflammatory mediators, such as cytokines (TNF-α, IL-6, IL-8), chemokines (CCL2, CCL5, CCL7), and other pro-inflammatory proteins (ICAM-1) [3]. These mediators serve to activate and attract leukocytes to the site of respiratory infection/inflammation. Release of cytokines by leukocytes then stimulates the airway epithelium to release more inflammatory mediators, thus making these cells central players in both the induction as well as perpetuation of the inflammatory response.

One of the major stimulants of the airway epithelium is TNF-α, which induces the production of numerous cytokines, chemokines, and other factors. TNF-α stimulation can activate NF-κB, MAP kinases, and induce the production of ROS, which can act as second messengers or oxidants of proteins and nucleic acids to potentiate NF-κB effects and inflammatory mediator production [4,5]. TNF-α can also act post-transcriptionally to promote inflammation by increasing the stability of cytokine mRNA [6]. We previously demonstrated that the RNA-binding protein HuR has widespread pro-inflammatory effects on the airway epithelium, mediated by the stabilization of cytokine mRNA after TNF-α treatment [7]. Thus, TNF-α treatment of human airway cells serves as an effective model to study regulation of the inflammatory response via a number of mechanisms and to test putative anti-inflammatory molecules.

There are currently few treatment options for inflammatory lung diseases such as severe asthma and COPD. Methoxyphenolic compounds have been shown to have anti-inflammatory action in leukocytes and endothelial cells, and we have previously shown the anti-inflammatory effects of apocynin (4-hydroxy-3-methoxy-acetophenone) in these cells [8]. Apocynin, a naturally occurring methoxyphenolic compound isolated from the medicinal plant *Picrorhiza kurroa*, has been described as a traditional medicinal treatment in numerous diseases, including asthma [9]. Its mechanism of action is not well understood, and in phagocytes it may act by inhibiting the translocation of the p47phox protein to the cell membrane, inhibiting formation of the ROS-generating NADPH oxidase complex. The resulting inhibition of ROS generation may play a crucial role in the attenuating the inflammatory response. We previously demonstrated that apocynin forms a dimer in the presence of ROS and peroxidase, and this dimer, diapocynin, is the active form of the drug [8]. It is thought the redox properties of the compound may promote oxidation of cysteine residues to alter protein structure and/or phosporylation, which may underlie the inhibitory effect on p47phox translocation [10]. Alternative models, particularly in the endothelium, suggest that apocynin may act as a free radical scavenger and its actions may not be related to NADPH oxidase inhibition [11].

The effect of apocynin and other methoxyphenols have not been well studied in epithelial cells. However, as these cells play a central role in the inflammatory response, we hypothesized that they are a target of the anti-inflammatory effect of methoxyphenols. In this study, we demonstrated the anti-inflammatory activity of apocynin, diapocynin, resveratrol, and multiple 4-substituted methoxyphenolic compounds in TNF-α-stimulated human airway cells. We showed that this effect is not mediated though inhibition of ROS generation or NF-κB inhibition, indicating that these compounds act via a distinct mechanism in airway epithelial cells. We also demonstrate that apocynin inhibits HuR binding to mRNA, suggesting a role in post-transcriptional regulation. The significance of these findings is discussed herein.

## Methods

### Cell culture and treatment conditions

The A549 and BEAS2B cell lines were obtained from American Type Culture Collection (ATCC, Manassas, VA), and cultured according to their recommended protocols. All cells were cultured at 37°C in humidified air containing 5% CO_2_. In short, A549 cells were maintained in F-12 K media (ATCC) containing 10% fetal bovine serum (Thermo Scientific, Rockford, IL), penicillin (100 U/mL) (Mediatech Inc., Manassas, VA), and streptomycin (100 mg/mL) (Mediatech). A549 cells were used from passages 3 to 35. BEAS2B cells were maintained in F12/DMEM (Life Technologies/Invitrogen, Frederick, MD) containing 10% FBS, penicillin (100 U/mL), and streptomycin (100 mg/mL). BEAS-2B cells were used from passages 30-45. Normal human primary bronchial epithelial cells were obtained from Lonza (Walkersville, MD), and cultured in BEGM media per manufacturer's recommendations. All experiments were performed between passages 4-6.

**Figure 1:**
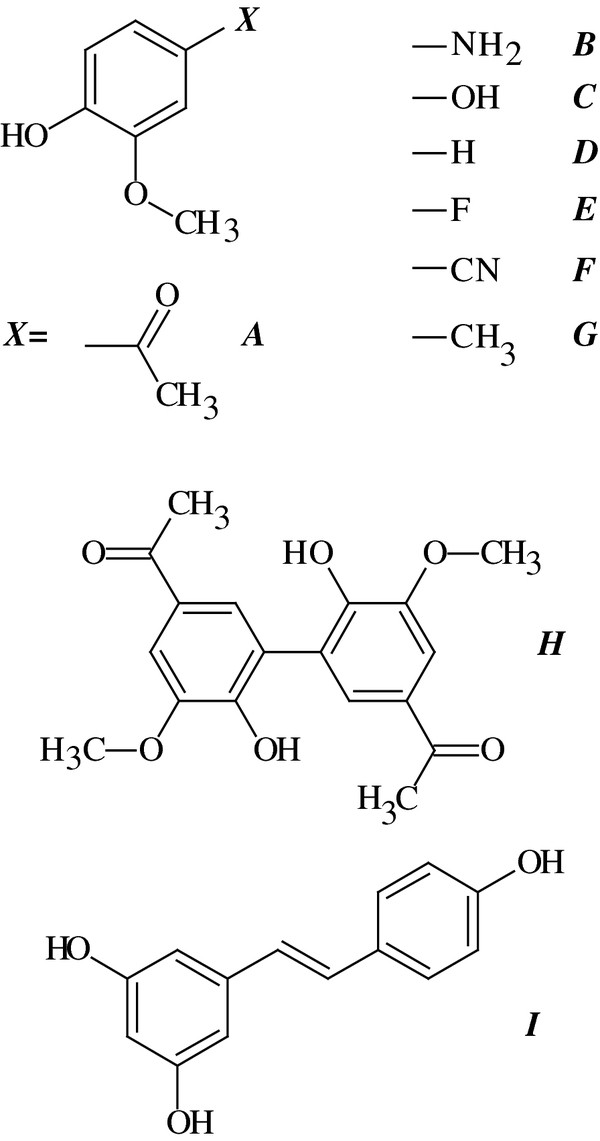
**Structures of compounds investigated in this study**. ***A ***- Apocynin (Apo), ***B ***- 4-amino-2-methoxyphenol (Amino), ***C ***- 2-methoxyhydroquinone (Hydroxyl), ***D ***- Guaiacol (Guai), ***E ***- 4-fluoro-2-methoxyphenol (Fluoro), ***F ***- 4-hydroxy-3-methoxybenzonitrile (Cyano), ***G ***- 4-methyl-2-methoxyphenol (Methyl), ***H ***- Diapocynin (Diapo), ***I ***- Resveratrol (Res).

Tumor necrosis factor alpha (TNF-α) solution was prepared by resuspending lyophilized protein (Becton, Dickinson and Company, Franklin Lakes, NJ) in 0.1% BSA (Bovine Serum Albumin) in ddH_2_O to give a working stock of 10 ng/uL. Cells were treated with 50 ng/mL of TNF-α as indicated [12]. To assess the anti-inflammatory effects of various compounds (Figure 1A-I), cells were treated with each compound concurrently with TNF-α, in the presence or absence of 0.5 mg/ml Horseradish Peroxidase (HRP) (Sigma Aldrich, St Louis, MO) and 25 μM H_2_O_2 _(Sigma Aldrich, St Louis, MO). The methoxyphenolic compound derivatives (Figure 1) were prepared by dissolving/diluting each separately in 100% ethanol to give stock solutions of 100 mM. Compounds A, C-G and I were purchased from Sigma Aldrich and compound B was purchased from Santa Cruz Biotechnology (Santa Cruz, CA). Compound H (Diapocynin) was synthesized as previously described [13,14]. Diluent (1% ethanol) was added to all untreated cells and TNF-α-treated cells.

### RNA isolation and real time PCR analysis of gene expression

Total cellular RNA was isolated from cell cultures using the TRIzol reagent method (Invitrogen). Cells were treated for 24 hours with 50 ng/mL of TNF-α in the presence of absence of 1 mM of each compound for the specified time. The concentration and quality of the isolated RNA was determined on an Ultrospec 3000 Spectrophotometer (Pharmacia Biotech). Cellular RNA (2 μg) was reversed transcribed to form total cellular cDNA via a High-Capacity cDNA Reverse Transcription Kit (Applied Biosystems). The cDNA levels of individual cytokines were assayed by using specific primer sets (Table 1) and a Sensimix SYBR green probe (Bioline) on a MYiQ2 real time PCR (Bio-Rad, Hercules, CA). All samples were run in triplicate and quantified using the comparative cycle threshold (C_T_) method [15,16].

**Table 1:** Primers used in qPCR

Gene of Interest	Primer
**CCL5 - Forward**	5'-TCCCCATATTCCTCGGACAC-3'
**CCL5 - Reverse**	5'-TGTACTCCCGAACCCATTTC-3'

**MIF - Forward**	5'-CCGGACAGGGTCTACATCA-3'
**MIF - Reverse**	5'-ATTTCTCCCCACCAGAAGGT-3'

**IL-6 - Forward**	5'-AGGAGACTTGCCTGGTGAAA-3'
**IL-6 - Reverse**	5'-CAGGGGTGGTTATTGCATCT-3'

**CCL7 - Forward**	5'-TGCTCAGCCAGTTGGGATTA-3'
**CCL7 - Reverse**	5'-GTCCTGGACCCACTTCTGTG-3'

**IL-8 - Forward**	5'-TAGCAAAATTGAGGCCAAGG-3'
**IL-8 - Reverse**	5'-AAACCAAGGCACAGTGGAAC-3'

**Serpin E1 - Forward**	5'-CTCTCTCTGCCCTCACCAAC-3'
**Serpin E1 - Reverse**	5'-GGAGAGGCTCTTGGTCTGAA-3'

**CXCL1 - Forward**	5'-CAAACCGAAGTCATAGCCAC-3'
**CXCL1 - Reverse**	5'-CTTCAGGAACAGCCACCAGT-3'

**GMCSF - Forward**	5'-GCTGCTGAGATGAATGAAACAG-3'
**GMCSF - Reverse**	5'-ACAGGAAGTTTCCGGGGTT-3'

**CCL11 - Forward**	5'-CCAGCTTCTGTCCCAACC-3'
**CCL11 - Reverse**	5'-AGTTTGGTCTTGAAGATCACAGC-3'

**CCL2 - Forward**	5'-GCTCAGCCAGATGCAATCA-3'
**CCL2 - Reverse**	5'-AGATCTCCTTGGCCACAATG-3'

**CXCL10 - Forward**	5'-CTGTACGCTGTACCTGCATCA-3'
**CXCL10 - Reverse**	5'-GGAGATCTTTTAGACCTTTCCTTG-3'

**GAPDH - Forward**	5'-GAGTCAACGGATTTGGTCGT-3'
**GAPDH - Reverse**	5'-TTGATTTTGGAGGGATCTCG-3'

### hCCL2, hCCL11 and GM-CSF ELISAss

Colorimetric human CCL2 (human chemokine (C-C motif) ligand 2), CCL11, and GM-CSF (granulocyte-macrophage colony stimulating factor) ELISAs (R&D Systems, Minneapolis, MN) were used per the manufacturer protocol, with one modification: the supplied wash buffer was replaced with 1X TBS-T (20 mM Tris pH 7.5, 150 mM NaCl, 0.1% Tween 20) to wash between steps. Prior to treatment, the growth media was removed from the adherent cells, PBS was used to wash once, and fresh serum-free media was added. Cells were mixed with TNF-α in the presence or absence of specific compounds or no treatment as described above for 24 hours. The media was collected, and secreted cytokines were measured by ELISA at a 1:40 dilution in ddH_2_O. The colorimetric absorbance was read at 450 nm using a GENios plate-reader (Tecan) running Magellan 6.6 software. All experiments were performed at least in triplicate. Data were analyzed using GraphPad Prism 4.0 (GraphPad) to generate the concentration-response curves and IC_50 _values were extrapolated.

### Human cytokine array and analysis

To measure expression of other epithelial-derived cytokines, the Proteome Profiler Human Cytokine Array Kit, Panel A (R&D Systems) was utilized per the manufacturer instructions. Cells were treated with TNF-α in the presence or absence of apocynin, or untreated, for 24 h and media was collected and incubated with the array membrane. Washes and treatments were performed without deviation from the recommended protocol. Membranes were treated with HyGlo chemiluminescence detection reagent (Denville Scientific, Metuchen, NJ) and exposed to film to for various time points to detect the signal. Films were scanned using a Canon Lide 100 instrument and subjected to densitometric analysis with ImageJ software (http://rsb.info.nih.gov/ij/). All experiments were carried out in triplicate.

### Reactive Oxygen Species (ROS) assay

Reactive oxygen species were detected with 3',6'-diacetate-2',7'-dichlorofluorescein (DCFH-DA) treatment. A 1 mM stock solution of DCFH-DA was prepared in DMSO (Sigma), and added to cells were treated in culture medium at a final concentration of 10 μM. A549 or BEAS2B cell cultures were first treated with solvent (No Tx - ethanol and H_2_O), TNF-α (50 ng/mL), or TNF-α and 1 mM of Compounds A-C, H, and I (separately) for 1 hour after the growth media was replaced with fresh media. The cultures were then treated with 10 μM DCFH-DA or diluent for 1 h. DCFH-DA fluoresces only after internalization in cells and conversion to 2', 7'-dichlorofluorescein which reacts with reactive oxygen species to produce fluorescence [17]. The 6 well plates in which the cells were grown are then placed in the GENios plate reader and the fluorescence was recorded (Ex - 485 nm; Em- 534 nm). The samples were all run in triplicate and plotted against TNF-α response.

### Assay for NFκB activation

A549 cell cultures were treated with diluent (1% ethanol and H_2_O), TNF-α (50 ng/mL), or TNF-α and 1 mM of apocynin for 1 hour. Cells were then harvested via scraping and lysed with PLB (Polysomial Lysis Buffer; 100 mM KCl, 5 mM MgCl2, 10 mM Hepes pH 7.0, 0.5% NP-40) for 10 minutes on ice. Isolation of nuclear lysates was performed as previously described [7]. The lysate was then loaded onto a 10% polyacrylamide-SDS gel and ran at 45 mA for 1 hour. The protein was transferred via semi-dry transfer to a nitrocellulose membrane. The membrane was probed with a primary antibody for NFκB/p65 (N-term), NFκB/p65 (Ser 311 phosphorylation) (Santa Cruz) and β-tubulin (Abcam, Cambridge, MA) as a loading control. The primary antibodies were recognized with a secondary antibody specific for rabbit (Jackson ImmunoResearch, West Grove, PA) after a 24 h incubation in 3% BSA/TBS-T. The membrane was then developed as described previously under the cytokine array. The experiment was run in triplicate and analyzed with ImageJ software.

### Biotin-RNA affinity isolation assay

A549 cell cultures were treated with diluent (1% ethanol) or TNF-α (50 ng/mL) in the presence or absence of 1 mM of apocynin. All treatments are for 24 hours except for apocynin treatment of lysed cells. In this case, cells were treated for 24 h with TNF-α, lysed, and treated with apocynin for 30 min. After cellular lysis with PLB, 1 μM of biotinylated CCL2 3'UTR RNA (synthesized as previously described) was added to the lysate for 30 minutes at room temperature in TENT buffer (10 mM Tris (pH 8.0), 1 mM EDTA, 250 mM NaCL, 1% v/v Triton X-100) and RNase Out [18]. The mixture was then exposed to magnetic beads coated with streptavidin (Dynabeads, Invitrogen) for 30 minutes at room temperature. The beads were prepared per manufacturer's instructions. Unbound protein was removed by washing three times with 250 μl of PBS. Protein was eluted with SDS-PAGE sample buffer (1 M Tris pH 6.8, 40% glycerol, 10% SDS, 1%bromophenol blue, H_2_O and 1 mM DTT) and then resolved on a 10% polyacrylamide-SDS gel and developed as previously described with a primary mouse antibody for HuR (Santa Cruz Biotechnology) [7].

### Statistical analysis

All experiments were performed in at least triplicate. Data were analyzed using either Excel 2007 (Microsoft) or GraphPad Prism 4.0. Values of significance (*p *≤ 0.05) were determined by non-parametric ANOVA testing between treatment conditions and controls. Error bars on all graphs represent the standard error of the mean.

## Results

### Anti-inflammatory effects of apocynin on human airway cells

**Figure 2:**
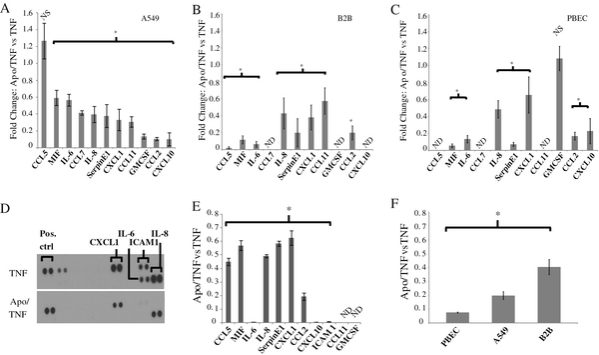
**Effects of apocynin on cytokine mRNA and protein levels under inflammatory conditions**. ***A***, Real time PCR data gathered from mRNA isolated from A549 cells treated with TNF-α (50 ng/ml) in the presence of absence of Apocynin (Apo, 1 mM) for 24 h. Shown are relative levels of inflammatory mediator mRNA in the Apo/TNF-α vs. TNF-α treatment (n = 3; *,*p *< 0.05; NS, not statistically significant. Cytokine gene expression changes were also measured in BEAS2B cells ***(B) ***and PBECs ***(C)***, ***D*,**Representative raw data from the protein array experiment, comparing cytokine levels in the Apo/TNF-α vs. TNF-α treatment. ***E***, ELISA and densitometeric analysis of cytokine protein array data, comparing cytokine levels in the Apo/TNF-α vs. TNF-α treatment (n = 3; *, *p *< 0.05; ND, not detected). ***F***, CCL2 expression was measured by ELISA in A549 cells, BEAS2B cells, and PBECs under the treatment conditions described above.

The respiratory epithelium is a major producer of cytokines and other inflammatory mediators in a variety of inflammatory lungs diseases [19]. As methoxyphenolic compounds have demonstrated anti-inflammatory effects, we sought to determine whether they attenuate the production of inflammatory mediators in human airway cells. A549 human airway cells were treated with TNF-α (50 ng/mL) to stimulate inflammatory mediator production, and the effect of apocynin was assessed by co-treatment with TNF-α with 1 mM apocynin (Compound A, Figure 1) for 24 hours. Expression of a panel of epithelial-derived cytokines known to be induced by TNF-α (Figure 2A) was assessed by qPCR. The ability of apocynin to inhibit the TNF-α stimulation of each cytokine is shown in Figure 2A as the ratio of the fold increase for each cytokine under Apocynin/TNF-α treatment compared to TNF-α treatment alone. Apocynin significantly attenuated the expression (*p *< 0.05, n = 3) of all of the cytokines measured by at least 50%, except for CCL5, whose mRNA expression was unchanged. The greatest effect was seen on GMCSF, CXCL10, and CCL2 where there was a 90% reduction in the level of TNF-α-induced mRNA production. Effects of apocynin on TNF-α-induced cytokine production was also measured in BEAS2B cells (Figure 2B) and normal human primary bronchial epithelial cells (PBECs, Figure 2C) to ensure that the results were not cell line specific. Apocynin exerted a similar anti-inflammatory effect, and a similar inhibitory effect was observed on the gene expression of numerous inflammatory mediators (IL-6, IL-8, CCL2, CXCL1, MIF, and Serpin E1).

As levels of mRNA and protein do not always correlate, and because many of these cytokines are post-transcriptionally regulated, we monitored the effect of apocynin on the protein levels of these cytokines [20]. A549 cells were treated as described above and secreted cytokine expression was analyzed by a combination of ELISAs and a cytokine array. Figure 2D depicts a representative image of the raw cytokine array data, with changes in IL-6, IL-8, ICAM-1, and CXCL1 shown between TNF-α and apocynin/TNF-α treatment. Densitomteric analysis was performed with ImageJ software and summarized in Figure 2E. For cytokines not present on the array, or in cases where the array failed to detect any cytokine production after TNF-α treatment, ELISAs were performed. Apocynin treatment leads to an almost complete loss of signal for ICAM-1 and IL-6, and a statistically significant (*p *< 0.05) inhibition of all other cytokines measured. CCL11 and GM-CSF expression could not be detected by either array or ELISA, indicating that these cytokines are poorly expressed in these cells after TNF-α treatment. Interesting, apocynin also inhibited the production of CCL5 protein, despite not having an effect on its mRNA level, suggesting that there is a post-transcriptional mechanistic component of the drug. Along the same lines, there was approximately a 50% change in IL-6 mRNA level with apocynin-treatment, but an almost complete inhibition in protein expression. For other cytokines (i.e. CCL2, IL-8) the change in protein and mRNA levels correlated well. We also measured the ability of apocynin to inhibit CCL2 protein production in TNF-α-stimulated BEAS2B and PBEC cells. Both cell types demonstrated similar effects as A549 cells (Figure 2F).

### Kinetics of apocynin effect on TNF-α-induced cytokine production

**Figure 3:**
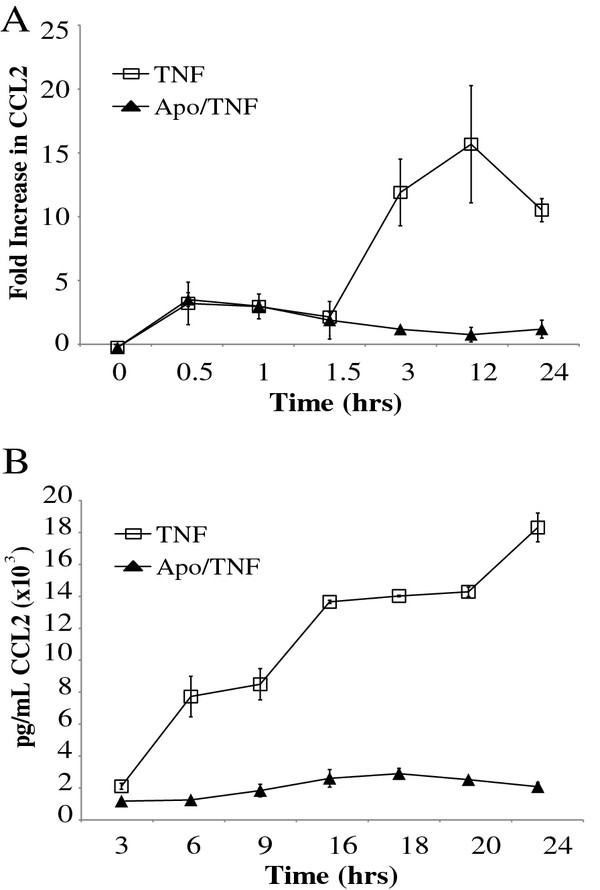
**Time course of apocynin effects on CCL2 protein and mRNA levels**. ***A***, CCL2 mRNA levels by real time PCR for TNF-α or TNF-α/Apocynin treatment in A549 cells for the indicated time (n = 3). ***B***, The effect of apocynin treatment on CCL2 protein levels for the indicated times after treatment with TNF-α or TNF-α/Apocynin (n = 3).

To determine the time course of apocynin inhibition on TNF-α-induced cytokine production, a kinetic assessment was performed at various time points. The greatest effects of TNF-α on CCL2 mRNA production begins between 1.5 and 3 h and peaks at 12 h (Figure 3A). The inhibitory effects of apocynin begin between 1.5 and 3 h and continue for 24 h. A similar effect is noted for CCL2 protein production. At 3 hours apocynin has begun to inhibit CCL2 protein production, and the effect is maintained at 24 hours levels (Figure 3B).

### Comparison of the anti-inflammatory effects of apocynin and diapocynin

We previously demonstrated that apocynin forms a dimer (Figure 1H) under oxidative conditions, and this dimer was the active form of the drug [8]. Dimerization required the presence of H_2_O_2 _and a peroxidase (such as myeloperoxidase), and these enzymes are not expressed in airway epithelial cells [21]. It is possible that under inflammatory conditions in vivo, phagocytic myeloperoxidases could induce dimerization of the drug, which could then be taken up by epithelial cells. However, it is also possible that the mechanism of action is different in the airway, and the monomer is active. To determine whether the apocynin monomer or dimer is the active form in airway epithelial cells, we analyzed the effect of apocynin in the presence or absence of H_2_O_2 _and horseradish peroxidase and diapocynin on CCL2 production in TNF-α treated cells.

**Figure 4:**
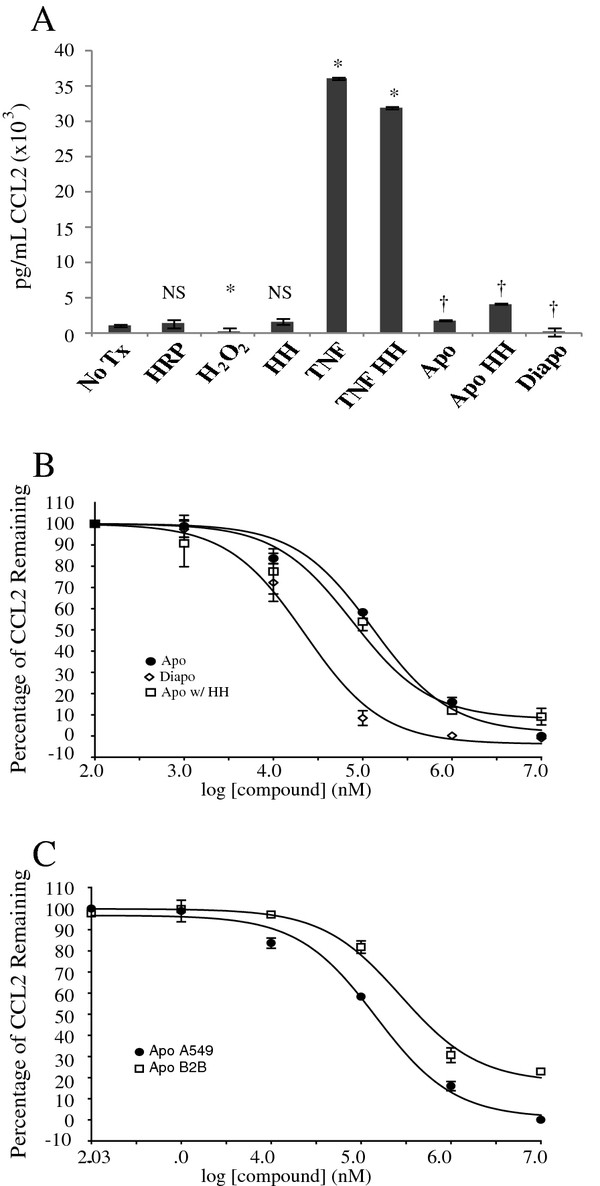
**Effect of apocynin and diapocynin on CCL2**. ***A***, effects of various treatments on CCL2 production: H_2_O_2_, HRP, H_2_O_2 _+ HRP (HH), TNF-α, apocynin (Apo), diapocynin (Diapo). All experiments were performed in triplicate; *, *p *< 0.05 when compared no treatment; †, *p *< 0.05 when compared to TNF-α treatment; NS, not statistically significant. ***B***, concentration-response curve of diapocynin and apocynin in the presence or absence of H_2_O_2 _+ HRP on CCL2 protein levels (n = 3). ***C***, concentration dependence of apocynin on CCL2 production was measured in BEAS2B cells and compared to effects in A549 cells.

By ELISA, CCL2 levels were unaffected by H_2_O_2_, HRP, and the combination of the two (Figure 4A). TNF-α treatment resulted in a 36 ± 6.5 fold increase in the amount of secreted CCL2 protein after 24 hours (*p *< 0.05, n = 3). Apocynin effectively prevented CCL2 production, reducing the levels to that of the no treatment control (*p *< 0.05, n = 3). Apocynin treatment in the presence of H_2_O_2 _and HRP similarly reduced CCL2 production. Diapocynin (1 mM) was the best inhibitor of CCL2, reducing levels to a point below the baseline level in unstimulated cells. The addition of HRP, H_2_O_2_, or the combination of the two had no effect on CCL2 level.

These data suggest that apocynin monomer also has anti-inflammatory activity, independent of the presence of HRP and H_2_O_2_. However, as there could be a difference in potency between the monomer and dimer, we measured the inhibitory effect on CCL2 production as a function of the concentration of each compound. A concentration-dependent effect was seen for the apocynin in the presence and absence of ROS and diapocynin (Figure 4B), without any toxicity to the cells observed at the highest concentration (10 mM). The following IC_50 _values for calculated each compound: 20.3 ± 3.8 μM for diapocynin, 103.4 ± 13.6 μM for apocynin with H_2_O_2 _and HRP, and 146.1 ± 5.2 μM for apocynin alone (Table 2). We also measured the IC_50 _for apocynin in BEAS2B cells, which 275 ± 5 μM, similar to that obtained for A549 cells (Figure 4C).

**Table 2:** Comparison of IC_50 _Values For Selected Compounds

Anti-Inflammatory Compound	IC_50 _(μM)
**Apocynin (A)**	146.1 ± 5.2

**Apocynin (A) with H_2_O_2 _and HRP**	103.4 ± 13.6

**Diapocynin (H)**	20.3 ± 3.8

**Resveratrol (I)**	42.7 ± 1.8

**Amino (B)**	410.0 ± 77.2

**Hydroxyl (C)**	64.3 ± 9.5

### Anti-inflammatory effects of other methoxyphenols in human airway cells

**Figure 5:**
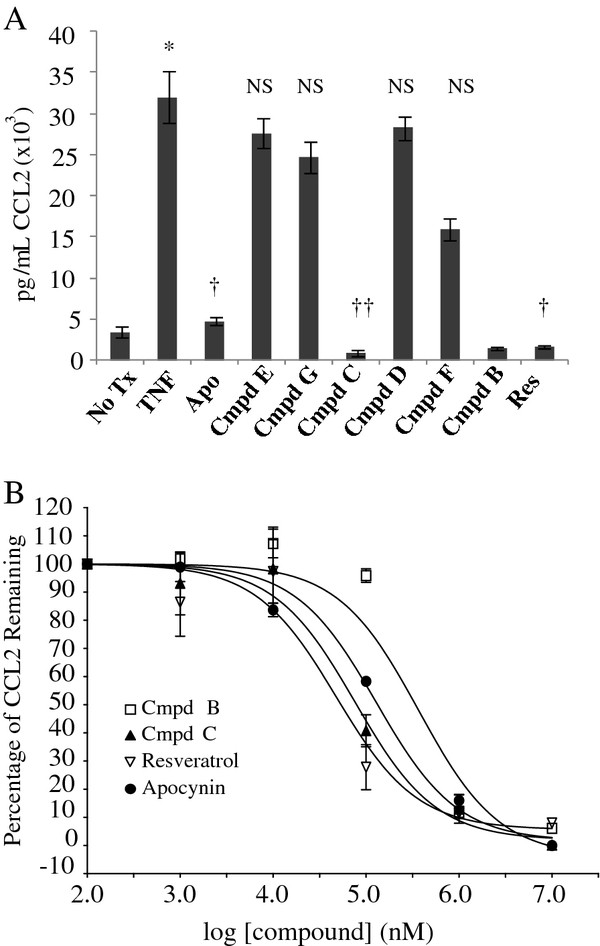
**Anti-inflammatory effect of compounds on CCL2 production**. ***A***, effect of 4-substituted methoxyphenolic compounds and resveratrol (Res) on TNF-α induced CCL2 production (n = 3; *, *p *< 0.05 when compared no treatment; †, *p *< 0.05 when compared to TNF; NS, not significant). ***B***,dose-response curves of various compounds on CCL2 production (n = 3).

In order to determine whether other methoxyphenols may also exhibit anti-inflammatory effects on human airway cells, the effects of various 4-substituted compounds and resveratrol were analyzed. As a screen, the effect of each compound at a concentration of 1 mM on TNF-α induced production of CCL2 was measured. Similar to previous experiments, apocynin shows a significant (*p *< 0.05, n = 3) reduction in amount of protein produced (Figure 5A). Compounds D, E, G show little effect on the amount of CCL2 protein produced while compounds B and C and I show an effect equal to or greater than apocynin (*p *< 0.05). For the latter compounds, varying concentrations were used to obtain a dose response curve for CCL2 production. Resveratrol (Compound I) had the lowest IC_50 _of 42.7 μM, followed by the hydroxyl derivative (Compound C) of 64.3 μM (Table 2). Both of those compounds have IC_50 _values lower than apocynin but higher than diapocynin. The amino derivative (Compound B) had the highest IC_50_, 410.9 μM.

### Mechanism of apocynin action

**Figure 6:**
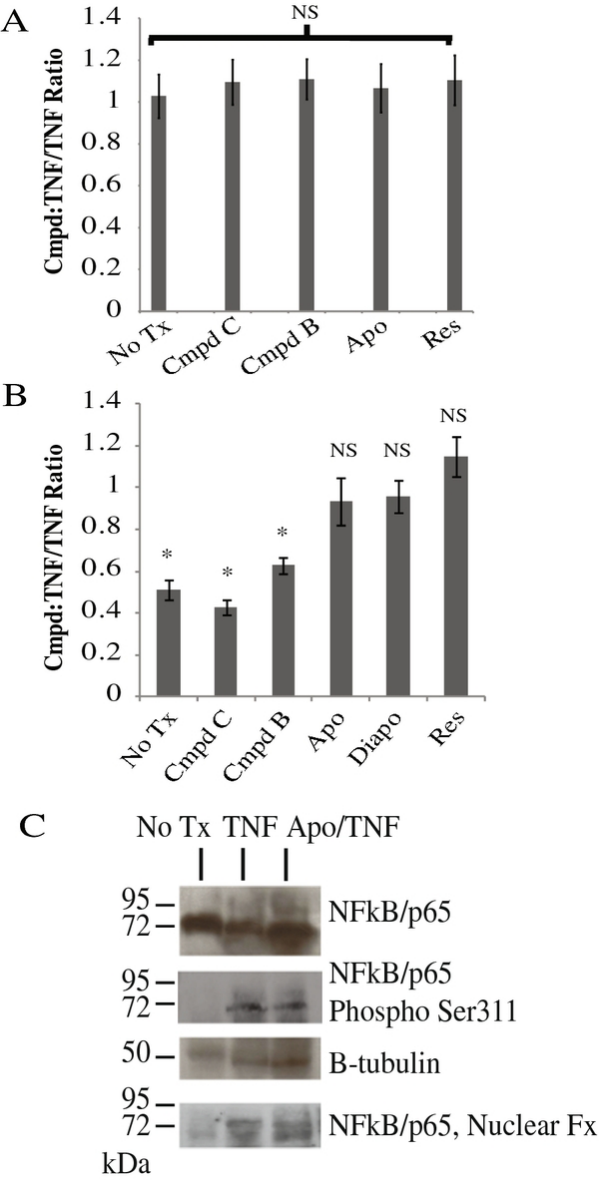
**Effect of compounds on ROS production and NF-κB activation**. DCFH-DA fluorescence assay various compounds after a 1 h treatment with TNF-α or TNF-α/Compound (1 mM). (*, *p *< 0.05 of TNF-α/compound vs TNF-α) in A549 ***(A) ***or BEAS2B ***(B) ***cells. ***C***, effect of TNF-α or TNF-α/Apocynin on expression, phosphorylation (at Ser311), or nuclear translocation of NF-κB. Data shown is representative of n = 3.

Apocynin was previously shown to inhibit production of ROS, either as an anti-oxidant and/or as an inhibitor of the p47phox component of NADPH oxidase complexes [22]. To determine whether apocynin inhibited ROS production, a fluorescent assay was utilized to quantitatively measure levels. Human airway cells (A549), untreated or treated with TNF-α (50 ng/ml) in the presence or absence of 1 mM of the compound, were then mixed with DCFH-DA (10 μM) for 1 h. Unexpectedly, DCFH-DA appeared to be toxic to the A549 cells, and approximately 50% of the cells were no-longer adherent to the tissue culture plate after 1 h. Fluorescence was monitored in these cells and the values are shown as a ratio relative to TNF-α treatment in Figure 6A. No difference was noted in ROS levels in any of the treatments, though it was not clear of these results were artifactual given the effect of DCFH-DA on the cells. We then repeated the experiment in BEAS2B cells (Figure 6B). In these cells, DCHF-DA did not appear to be toxic, and as expected, TNF-α induces ROS generation relative to the untreated cells. The hydroxyl and amino compounds significantly attenuated the ROS production, however, apocynin, diapocynin, and resveratrol had no effect on the levels. These data indicate that the inhibitory effect of these compounds on cytokine production does not correlate with their ability to reduce generation of ROS.

As most of the cytokines inhibited by apocynin are regulated by NF-κB, it is possible that the compounds might directly target the transcription factor. Apocynin has been proposed to oxidize proteins to alter phosphorylation or translocation, which are essential for NF-κB function [23,24]. The activation of NF-κB in A549 cells was assessed by phosphorylation at Ser311 and translocation to the nucleus after TNF-α treatment in the presence or absence of apocynin (1 mM). As expected, TNF-α stimulation induced the phosphorylation of NF-κB as well as its translocation to the nucleus (Figure 6C). However, apocynin had no effect on either process, indicating that it does not inhibit NF-κB.

**Figure 7:**
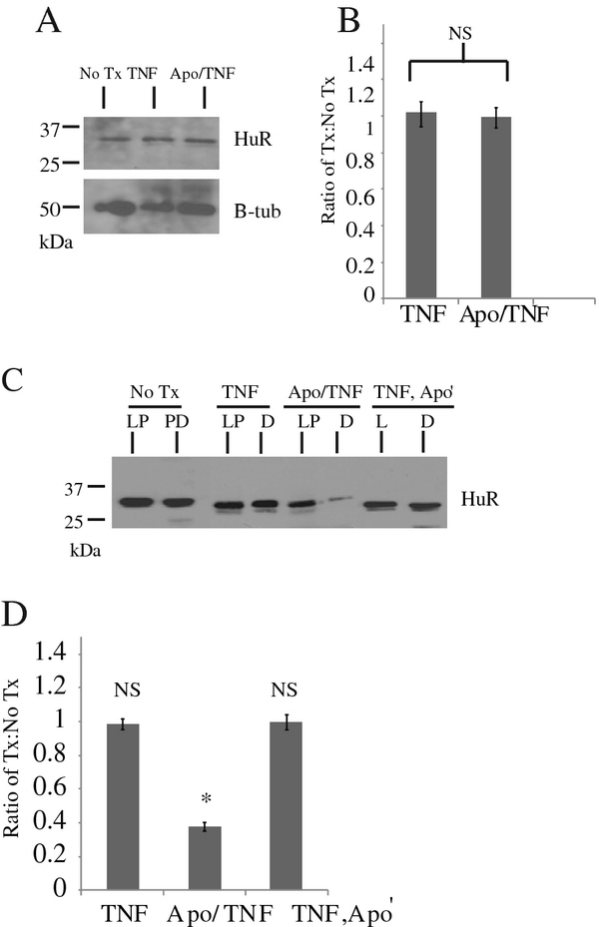
**Effect of apocynin on HuR binding to CCL2 RNA**. ***A***,HuR expression was monitored by Western blot under various treatment conditions: unstimulated, treatment of cells with TNF-α or TNF-α/apo for 24 h. Data is a representative blot of n = 3. ***B***, Quantitation of HuR protein level after various treatments relative to unstimulated cells by densitometry, using β-tubulin as a loading control for normalization. ***C***,binding of HuR to CCL2 3'UTR by biotin affinity isolation. Lysates were incubated with biotinyated CCL2 3'UTR RNA, mixed with streptavidin beads, washed, and isolated proteins were analyzed by Western blot to identify HuR binding. TNF,Apo', cells were treated with TNF-α for 24 hours, lysed and then treated with apocynin (1 mM) for 30 min. Blot of representative data of n = 4. Each lane was taken from the same exposure of the same Western blot and re-arranged in order for the figure (L- pure lysate, PD- lysate after treatment with beads and biotinylated RNA). ***D***, Densitometeric analysis of the Western blot data. (*, *p *< 0.05 when compared to TNF:No Tx Ratio), n = 4).

We previously demonstrated that TNF-α treatment led to the HuR-mediated mRNA stabilization of a number of prominent airway epithelial-derived cytokines and chemokines [7]. Post-transcriptional regulation is emerging as a central mechanism in controlling an inflammatory response, and as such we investigated the effects of apocynin on binding of HuR to CCL2. We previously demonstrated that HuR binds to the 3'UTR of CCL2 transcript, stabilizing the cytokine mRNA and increasing its expression [7]. HuR is expressed at baseline in A549 cells, and treatment with TNF-α in the presence or absence of apocynin did not significantly alter cytoplasmic HuR expression (Figure 7A, B). Association of HuR with the CCL2 3'UTR was assessed using a biotinylated RNA affinity isolation assay. Cells (unstimulated or treated with TNF-α in the presence or absence of apocynin) were lysed, mixed with biotinylated RNA, and RNA-protein complexes were isolated using magnetic streptavidin beads. Eluted protein was analyzed by Western blot, which demonstrated that apocynin treatment inhibited the binding of HuR (Figure 7C, D) [7]. If TNF-α-treated cells were lysed, then treated with apocynin, no effect on HuR binding was observed, indicating the effects of the drug required the presence of intact cells.

## Discussion

The airway epithelium plays a central role in the initiation and propagation of the inflammatory response. In response to inflammatory stimuli such as cytokines, airway epithelial cells produce large amounts of cytokines, chemokines and other inflammatory mediators. We investigated the anti-inflammatory effects and mechanisms of action of several 4-substituted methoxyphenols and resveratrol on TNF-α-stimulated human airway cells. The significance of these findings is discussed herein.

### Apocynin attenuate the inflammatory effects of TNF-α in airway epithelial cells

We and others have previously demonstrated that apocynin and other 4-substituted methoxyphenols have anti-inflammatory properties in endothelial cells as well as phagocytes, implicating them as potential therapeutic agents in vascular diseases [8,10]. Their effects on airway epithelial cells have not been characterized, however, even though apocynin has been used as a traditional anti-asthma agent, and there are multiple animal studies demonstrating anti-inflammatory effects in asthma [21,25]. Using a TNF-α-stimulated model of human airway epithelial cells, we demonstrated that apocynin attenuated the production of numerous important epithelial-derived cytokines and chemokines. For the majority of the cytokines investigated, apocynin significantly reduced their production by more than 50%. For some mediators, such as IL-6, CXCL10, and ICAM-1, apocynin treatment led to almost undetectable levels. Inhibition of IL-6, an inflammatory cytokine with widespread actions, would be expected to produce anti-inflammatory effects in a wide variety of settings. Furthermore, the CC chemokines (i.e. CCL2, CCL5) play key roles in the recruitment of monocytes, dendritic cells, and T-cells, while CXC chemokines (CXCL1, CXCL10, IL-8) are important neutrophil recruiters [26]. The cell adhesion molecule ICAM-1 also plays a key role in migration of leukocytes, and together these data indicate that apocynin would be effective in inhibiting the trafficking of leukocyotes to sites of inflammation.

The concentration-dependence of apocynin on cytokine production was demonstrated for the inhibition CCL2. This was chosen as a model cytokine as it is highly expressed in the respiratory epithelium, is an important chemoattractant for monocytes, dendritic cells, T-cells, and basophils [27], and is significantly attenuated by apocynin treatment. Apocynin was found to have a concentration-dependent effect, and an IC_50 _value of 146 μM was calculated. Though this value was somewhat high, apocynin has been shown to have an excellent safety profile in rodents, with an LD_50 _of 9 g/kg, and we observed no toxic effects on our cells even at concentrations of 10 mM [9]. These findings were echoed in a phase I human study, where 6 ml of a 0.5 mg/mL of apocynin was nebulized into the airway of normal subjects without any adverse effects [28].

### Effects of other 4-substituted methoyphenols and resveratrol

To identify more potent anti-inflammatory compounds, we measured the effects of other 4-substituted methoxyphenols and the related compound resveratrol on production of inflammatory mediators. In general, we found that compounds with more electron withdrawing groups were more likely to have anti-inflammatory effects. Of the compounds tested, the hydroxyl-substituted compound (compound C) had the lowest IC_50 _of the monomeric compounds tested. As the apocynin dimer was the most potent compound, it is possible that the dimer of the hydroxyl-substituted compound would also have enhanced activity relative to the monomer, and this might be explored as strategy to produce a more potent anti-inflammatory agent. Resveratrol is a closely related compound that has been suggested to have benefit in a wide variety of diseases, including inflammatory diseases [29]. In this study, it behaved similarly to the methoxyphenols, and had potency similar to diapocynin. These data demonstrate that resveratrol has anti-inflammatory properties in human airway cells supports its therapeutic potential in treatment of asthma and COPD. Somewhat surprisingly, the fluorinated compound (compound E) showed no anti-inflammatory activity, despite having better efficacy over apocynin in inhibiting ROS production in neutrophils and mononuclear leukocytes [10]. This suggests that the mechanism of action of apocynin in epithelial cells is distinct from its action in leukocytes, and this concept is explored in more detail below.

### Is diapocynin the active form of the compound?

Previously, we showed that apocynin forms a dimer in the presence of reactive oxygen species and peroxidase, and that the dimer was the active form of the drug [8,30]. In phagocytes, apocynin is taken up by the cell and converted to the dimeric form in the presence of ROS and myeloperoxidase [30,31]. To investigate the activity of the monomer and dimer, we treated TNF-α-challenged cells with apocynin, apocynin in the presence of ROS and exogenous peroxidase, and diapocynin. Diapocynin was found to have the lowest IC_50_, followed by apocynin plus ROS and peroxidase, then apocynin alone. As peroxidases are not known to be expressed in epithelial cells, these data indicate that the monomer does have activity, though the dimer is more potent. The intermediate potency of the apocynin/ROS/peroxidase mix suggests that some dimer is formed under these conditions, and thus the effects seen may be due to a mix of monomer, dimer.

### Mechanism of action of apocynin in the airway epithelium

The observation that the apocynin monomer has activity independent of the dimer in airway epithelial cells suggests that it may act via a different mechanism of action than in phagocytes and endothelium. Apocynin has been proposed to inhibit ROS production by inhibiting the NADPH oxidase complex, or by acting as a free radical scavenger. Preventing formation of ROS generation may have anti-inflammatory effects via a variety of mechanisms, including the activation of important transcription factors such as NF-kB as previously reported [32]. In our system, we observed no correlation between ROS inhibition and anti-inflammatory activity. It should be noted that we did not specifically measure mitochondrial ROS generation, and it is not known if apocynin might inhibit this pathway specifically.

Apocynin treatment did not affect phosphorylation and translocation of the NF-κB transcription factor, suggesting that its anti-inflammatory effects were not due to effects on this transcription factor in epithelial cells. However, as nuclear translocation and Ser311 phosphorylation are not the only markers of NF-κB activity, we cannot completely exclude exclude that apocynin has effects on NF-κB activity or other transcription factors, and this will require further study.

The effect of TNF-α and other inflammatory mediators on gene regulation in epithelial cells is complex, and involves changes on the level of transcription as well as post-transcriptional effects. Post-transcriptional regulation is emerging as a crucial control point in the regulation of the inflammatory response, and most often involves changes on mRNA stability or translation. RNA-binding proteins, such as HuR, bind to AU-rich regions and other regulatory elements in the 3'UTR of inflammatory transcripts and promote mRNA stability or affect translation [33]. We previously demonstrated that TNF-α stimulation led to post-transcriptional up-regulation of many cytokines and chemokines, mediated by HuR stabilization of transcripts [7]. As shown in Figure 7C, D, apocynin inhibited the binding of HuR to CCL2 RNA, suggesting that at least some of its effects may be post-transcriptional in nature. As many of the other cytokines that apocynin repressed (i.e. IL-6, IL-8, CXCL1) are targets of HuR, a loss of HuR effect may also underlie these effects [7], We also observed that the effects of apocynin on mRNA and protein levels did not always correlate, supporting a role in post-transcriptional regulation. The molecular mechanisms of apocynin are not clear. Apocynin may act to oxidize cysteine residues on proteins to alter structure or post-translational modifications [10]. The effect on HuR binding required treatment of intact cells rather than a cell lysate, indicating that apocynin is not simply binding to the protein. It is possible that the drug acts upstream of HuR, such as on kinases or phosphatases that regulate HuR, or may act on other RNA-binding proteins that compete with HuR for mRNA-binding. HuR has been reported to be phosphorylated by a number of kinases, including Chk2, Cdk1, p38, PKCα and PKC δ [34]. In particular, Chk2, p38, and PKC's have been shown to affect binding to mRNA, and could be affected by apocynin. Future work will be aimed at determining the effects of apocynin on HuR and its overall effects on transcription and post-transcriptional control.

## Conclusions

Methoxyphenols represent a class of novel anti-inflammatory compounds that may have therapeutic implications in a variety of inflammatory diseases by acting on the epithelium as well as leukocytes.

## Abbreviations

3'UTR: 3' Untranslated region; BSA: Bovine serum albumin; CCL: Chemokine (C-C motif) ligand; CXCL: Chemokine (C-X-C motif) ligand; DCFH-DA: 3',6'-diacetate-2',7'-dichlorofluorescein; HRP: Horseradish peroxidase; ICAM: Inter-cellular adhesion molecule; IL: Interleukin; MIF: Macrophage migration inhibitory factor; PBEC: Normal human primary bronchial epithelial cells; qPCR: Real time polymerase chain reaction; ROS: Reactive oxygen species; TNF-α: Tumor necrosis factor alpha.

## Competing interests

The authors declare that they have no competing interests.

## Authors' contributions

KRH carried out all of the studies in the methods section, performed the analysis of collected data and drafted the manuscript. DKJ synthesized diapocynin and co-conceived of the study. FTI conceived of the study, participated in its design and coordination and helped in drafting of the final manuscript. All authors read and approved the final manuscript.
